# Oral Feeding Challenges in Children With Tracheostomy Can Improve Feeding Outcomes, Even With the Finding of Aspiration

**DOI:** 10.3389/fped.2019.00362

**Published:** 2019-09-04

**Authors:** You Gyoung Yi, Byung-Mo Oh, Seoyon Yang, Hyung-Ik Shin

**Affiliations:** ^1^Department of Rehabilitation Medicine, Seoul National University College of Medicine, Seoul, South Korea; ^2^Department of Rehabilitation Medicine, Seoul National University Hospital, Seoul, South Korea; ^3^Department of Rehabilitation Medicine, Ewha Women's University Seoul Hospital, Ewha Women's University School of Medicine, Seoul, South Korea

**Keywords:** children, deglutition, deglutition disorders, tracheostomy, videofluoroscopic swallowing study, aspiration, oral feeding

## Abstract

It has been suggested that oral feeding trial has therapeutic implications for improving oral-motor and swallowing function in infants and young children fed via an enteral tube or gastrostomy. This study aimed to investigate whether oral feeding challenges in children with tracheostomy could improve feeding outcomes, even with the finding of aspiration compared to those who did not receive oral feeding at all. Children (age <7 years) with tracheostomy who had thin fluid aspiration on videofluoroscopic swallowing study (VFSS) were included in this retrospective study. Enrolled children were then divided into two feeding method groups according to the physician's decision at the time of VFSS: oral feeding (OF) group and non-oral feeding (NOF) group. Data were obtained from 47 children (median age: 49.75 months, interquartile range [IQR]: 24.08–79.42). The incidence of pneumonia within 1 year after the VFSS was not different between NOF (*n* = 17) and OF (*n* = 30) groups. In OF group, 11 subjects achieved full oral feeding and 16 subjects were in partial oral feeding status 1 year after the VFSS. On the contrary, only one subject achieved full oral feeding and 5 subjects were in partial oral feeding status in NOF group (*p* < 0.001). Initial and follow-up penetration-aspiration scale on VFSS were different only in the OF group (*p* = 0.003). These results suggest that oral feeding challenges might be attempted even with the findings of aspiration in infants or young children with tracheostomy.

## Introduction

In children with swallowing difficulties, aspiration of food and fluid is commonly observed and is associated with a wide range of diseases. Current management decisions for aspiration generally include tube feeding, restricting aspirated diets, and providing texture-modified foods and thickened fluids (1–3). Young children usually refuse thickened fluids (3), resulting in a management dilemma for both medical professionals and families (2).

With regard to starting tube feeding during the first few years of life, it has been reported that the feeding outcome could be poor although the pharyngeal phase of swallowing is well preserved (4). Therefore, oral feeding during tube feeding is recommended and encouraged (4). It is reported that providing a taste or texture experience in early childhood, not only for nutritional purposes, can also help facilitate chewing skills in tube feeding children (5). Children who are exclusively tube fed would not have the opportunity to experience the sensations of food in the mouth and might be deprived of developing the oral-motor skills to manage different food consistencies and textures (6).

In 2016, McSweeney et al. reported that oral feeding of nectar or honey-thickened liquids instead of tube feeding via gastrostomy reduced the hospitalization frequency in children with aspiration or penetration findings on their videofluoroscopic swallow study (VFSS) (3). Prior to this report, we recommended exclusive tube feeding without oral feeding trial for children who showed aspiration findings on VFSS. However, based on the report and our clinical experience, we changed the strategy and began to try oral feeding challenges in infants or young children with aspiration instead of exclusive tube feeding. Especially in infants and young children with tracheostomy, oral feeding was tried more aggressively than before because the removal of aspirates could be possible through the tracheostomy (6). The amount of oral feeding challenges ranged from minimal to full nutrition according to the amount of aspiration, pharyngeal wall motion, epiglottis closure, and caregiver skills. A physician (one of the authors) decided whether to start oral feeding trial and the amount of the challenge.

In this study, we hypothesized that swallowing function could improve over time if infants or young children attempted oral feeding before the aero-digestive centers are fully established.

Thus, this study aimed to investigate whether oral feeding challenges in children with tracheostomy can improve feeding outcomes, even with the finding of aspiration compared to group that did not receive oral diets at all.

## Materials and Methods

All study-related procedures were performed in accordance with the ethical standards of the institutional and/or national research committee and the 1964 Declaration of Helsinki. Ethical approval for the study was obtained from our institutional review board (Approval No. 1803-115-932), which waived the requirement for informed consent due to the retrospective nature of the study. The following inclusion criteria were applied to potential subjects: (1) confirmed aspiration of fluid in a VFSS at <7 years of age between 2011 and 2017, (2) had a tracheostomy at the time of VFSS, and (3) medical records were available at least 1 year after the initial VFSS. When performing VFSS, foods with fluid consistency (e.g., water, juice) were always used, but foods with different consistencies (e.g., Yoplait, puree, cookies) were also used depending on individual eating habits. In many cases, parents brought foods that the children commonly ate at home and we tested these diets. VFSS results when using the diets other than fluid ones were not included in the inclusion or exclusion criteria. Patients with fluid swallowing of <2 times on VFSS records and those who refused food during VFSS were excluded from the analysis.

Enrolled children were then divided into two groups according to the feeding method received, per physician's decision at the time of VFSS: (1) oral feeding group (OF group), who were recommended partial oral feeding (POF) or full oral feeding (FOF) and (2) non-oral feeding (NOF) group, who were recommended exclusive tube feeding.

### Oral Feeding Trial

The caregiver played an important role in the oral feeding challenge. Oral feeding trial was conducted only when the parents were skillful and cooperative in suctioning during and immediately after the oral feeding. The oral feeding trial tended to be not applicable in school-age children, because parents were not able to perform suction while the children were in school. Therefore, we only included preschool children (<7 years of age) for the analysis. The oral diet was started at a low dose of 0.1–0.2 cc for the first time and gradually increased. The physician recommended the rate of increase considering the results of VFSS. Suction was carried out using portable equipment for home use. The depth and frequency of suctioning were not specified, but caregivers were instructed to perform shallow and gentle suctioning.

### Outcome Assessment

Primary outcome was defined as the feeding status (FOF, POF, or NOF) 1 year after the VFSS. The feeding status at the time and 1 year after the VFSS was recorded for all children through a medical record survey. Secondary outcomes included the occurrence of pneumonia and days of hospitalization related to pulmonary complications within 1 year after the VFSS.

### Patient Characteristics Assessed at the Initial VFSS

Patient records were reviewed for the sex and age of the patients at the time of their first VFSS. Initial feeding status before the VFSS was recorded for all children. The penetration-aspiration scale (PAS) on VFSS was rated by one of the authors. PAS scale is an 8-point scale developed to characterize the severity of airway invasion events viewed during VFSS, capturing the location to which material is observed to travel and then qualifying that information based on whether the material remains there at the end of the swallow or is ejected to safer (anatomically higher) locations (7). A score of 1 reflects no entry of material into the airway, scores of 2–5 reflect penetration of material past the laryngeal aditus into the supraglottic space and traveling as far as the true vocal folds, while scores of 6–8 reflect tracheal aspiration of material below the true vocal folds (7, 8).

### Follow-Up VFSS

For children who underwent both initial VFSS and 1 year follow-up VFSS, the pharyngeal transit time (PTT) as well as the PAS scale (7, 8) were compared to verify improvement or deterioration of swallowing function. PTT was defined as the total time of the bolus passage through the pharynx (9), from when the bolus head passed the ramus of the mandible to the time the bolus tail completely cleared the pharyngoesophageal segment (10). The PAS and PTT were rated retrospectively by one of the authors who were blinded to the group assignment.

### Statistics

Differences in continuous variables between the two groups were analyzed using the Wilcoxon rank sum test. In case of categorical variable, statistical significance was calculated by using Chi-square or Fisher's exact test. Main diagnoses were categorized as brain lesion as well as neuromuscular, cardiac, gastrointestinal, pulmonary, and otolaryngeal comorbidities for univariate and multivariate analyses; the comorbidities were not considered to be mutually exclusive. Given that the primary outcome comprised three categorical nominal variables (POF, FOF, and NOF), multinomial logistic regression was used to estimate the odds ratio (OR). Mann-Whitney U-test was used to compare initial PTT and follow-up PTT between groups. Analyses were performed using SAS statistical software (SAS system for Windows, version 9.4; SAS institute, Cary, NC). *p* < 0.05 was considered statistically significant.

## Results

### Subjects

**Figure 1:**
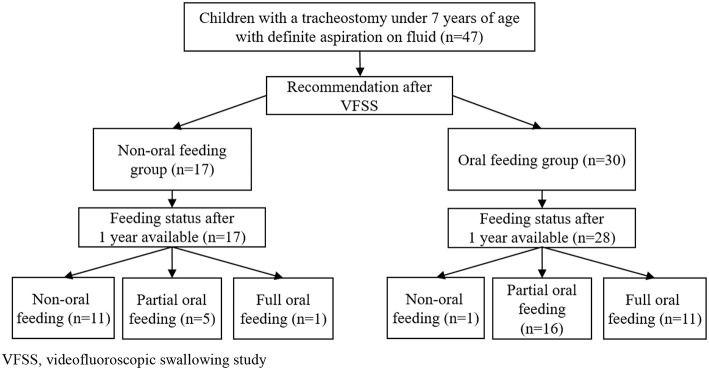
Flowchart of children with tracheostomy younger than 7 years of age with definite fluid aspiration confirmed via VFSS. VFSS, videofluoroscopic swallowing study.

Data were obtained for 47 children (median age: 49.75 months, interquartile range [IQR]: 24.08–79.42) who had tracheostomies and confirmed aspiration of fluid on initial VFSS between 2011 and 2017 (Figure 1). Seventeen children were assigned to the NOF group (median age: 61.75 months, IQR: 31.58–99.04), and 30 children to the OF group (median age: 35.42 months, IQR: 22.58–73.29). The characteristics of each group at the time of the VFSS are presented in Table 1. Initial feeding status before VFSS was not different between the OF and NOF groups (*p* = 0.152).

**Table 1:** Characteristics of subjects at the time of the videofluoroscopic swallowing study.

**Characteristics**	**NOF group (*n* = 17)**	**OF group (*n* = 30)**	**Total (*n* = 47)**	***p***
Female sex, *n* (%)	4 (23.53)	14 (46.67)	18 (38.30)	0.117^*^
Age, median (IQR), months	61.75 (31.58–99.04)	35.42 (22.58–73.29)	49.75 (24.08–79.42)	0.257^**^
Main diagnosis				
Myopathy/motor neuron disease	7 (41.18)	5 (16.67)	12 (25.53)	0.064^*^
Brain lesion	6 (35.29)	14 (46.67)	20 (42.55)	0.449^*^
Others Croup CATCH 22 syndrome Bronchopulmonary dysplasia VACTERL association Haddad syndrome Tracheoesophageal fistula Subglottic stenosis Treacher Collins syndrome Cleft palate	4 (23.53) 0 (0) 0 (0) 1 (5.88) 0 (0) 0 (0) 0 (0) 1 (5.88) 0 (0) 0 (0)	11 (36.67) 1 (3.33) 1 (3.33) 2 (6.67) 1 (3.33) 2 (6.67) 1 (3.33) 1 (3.33) 1 (3.33) 1 (3.33)	15 (31.91) 1 (2.13) 1 (2.13) 3 (6.38) 1 (2.13) 2 (4.26) 1 (2.13) 2 (4.26) 1 (2.13) 1 (2.13)	0.517^***^
CHARGE syndrome	2 (11.76)	0 (0)	2 (4.26)	
Initial feeding status, *n* (%)				0.152^***^
NOF	11 (64.71)	10 (33.33)	21 (44.68)	
Partial OF	4 (23.53)	13 (43.33)	17 (36.17)	
Full OF	2 (11.76)	7 (23.33)	9 (19.15)	
Initial PAS, median (IQR)	8 (8–8)	8 (8–8)	8 (8–8)	0.631^**^

p-values calculated from

* Chi-square test,

** Wilcoxon rank-sum test, or

*** Fisher's exact test, where appropriate.

*NOF, non-oral feeding; OF, oral feeding; IQR, interquartile range; PAS, penetration-aspiration scale*.

### Changes in Feeding Status

In children with tracheostomies, there was a significant difference in feeding status after 1 year between the OF and NOF groups (Table 2, Figure 1). In the OF group, 11 subjects achieved FOF and 16 subjects were in POF status. The incidence of pneumonia and pulmonary inpatient days within 1 year after the VFSS was not different between the OF and NOF groups.

**Table 2:** Outcome assessment 1 year after videofluoroscopic swallowing study in each group.

**Characteristics**	**NOF group (*n* = 17)**	**OF group (*n* = 30)**	**Total (*n* = 47)**	***p***
Feeding status after 1 year, *n* (%)				<0.0001^*^
NOF Partial OF Full OF	11 (64.71) 5 (29.41) 1 (5.88)	1 (3.57) 16 (57.14) 11 (39.29)	12 (26.67) 21 (46.67) 12 (26.67)	
Pneumonia presence within 1 year, *n* (%)	8 (47.06)	9 (30)	17 (36.17)	0.242^**^
Pulmonary inpatient days within 1 year, mean (SD)	5 (8.3)	3.93 (7.59)	4.32 (7.78)	0.304^***^

p-values calculated from

* Fisher's exact test,

** Chi-square test, and

*** *Wilcoxon rank-sum test. NOF, non-oral feeding; OF, oral feeding*.

The presence of neuromuscular disease was different between the two groups, with 8 (47.06%) in the NOF group and 5 (16.67%) in the OF group having a neuromuscular disease (*p* = 0.041). Brain lesions were present in 8 (47.06%) children in the NOF group and 15 (50%) in the OF group. Gastrointestinal, cardiac, otolaryngology, and pulmonary comorbidities were present in 2 (11.76%), 5 (29.41%), 3 (17.65%), and 11 (64.71%) children in the NOF group and 6 (20%), 10 (33.33%), 11 (36.67%), and 15 (50%) in the OF group, respectively. There were no differences in the main diagnosis categories, except for the neuromuscular disease category, between the OF and NOF groups.

Univariate and multinomial logistic regression analysis was conducted to calculate the OR of each factor affecting the feeding status after 1 year. Compared with the NOF group, the OF group showed an OR of 35.714 (*p* = 0.002) for feeding status 1 year after VFSS for POF compared with NOF (reference group), and an OR of 125 (*p* = 0.001) for FOF compared with NOF (Table 3). Various comorbidities including neuromuscular comorbidity and initial feeding status before the VFSS were not found to be significant predictors of dietary status 1 year after VFSS. To control multi-collinearity between factors with group variable (OF group vs. NOF group) included in the model, stepwise selection was performed (entry condition *p* < 0.05, removal condition *p* > 0.05). However, there was no factor at the significance level of 5% with group variable (OF group vs. NOF group) included in the model.

**Table 3:** Univariate analysis via multinomial logistic regression for feeding status (full oral feeding, partial oral feeding, or non-oral feeding) 1 year after the initial VFSS.

	**Feeding status after 1 year**	**Unadjusted OR**	**95% CI lower limit**	**95% CI upper limit**	***p***
Age (years)					0.9366
	NOF^a^	1.0			
	POF	0.924	0.595	1.435	0.7242
	FOF	0.938	0.57	1.543	0.8009
Group^*^					0.0022
	NOF	1.0			
OF vs. NOF group^*^	POF	35.714	3.597	333.33	0.0022
OF vs. NOF group^*^	FOF	125	6.711	>999.999	0.0012
Sex					0.8757
	NOF	1.0			
Female vs. male	POF	1	0.222	4.502	1.000
Female vs. male	FOF	1.429	0.271	7.518	0.6738
Initial feeding status					0.1408
	NOF	1.0			
OF vs. NOF	POF	2.2	0.504	9.611	0.2946
OF vs. NOF	FOF	6.0	1.018	35.374	0.0478
Neuromuscular					0.9031
	NOF	1.0			
No vs. yes	POF	1.25	0.271	5.765	0.7748
No vs. yes	FOF	1.5	0.254	8.844	0.6542
Brain					0.693
	NOF	1.0			
No vs. yes	POF	0.75	0.181	3.115	0.6921
No vs. yes	FOF	1.4	0.279	7.015	0.6824
Otolaryngeal					0.5287
	NOF	1.0			
No vs. yes	POF	1.067	0.205	5.543	0.9388
No vs. yes	FOF	0.467	0.082	2.656	0.3904
Respiratory					0.1914
	NOF	1.0			
No vs. yes	POF	0.7	0.162	3.023	0.6328
No vs. yes	FOF	2.8	0.532	14.735	0.2243
Cardiac					0.2192
	NOF	1.0			
No vs. yes	POF	2.5	0.572	10.932	0.2235
No vs. yes	FOF	5	0.753	33.213	0.0957
Gastrointestinal					0.9784
	NOF	1.0			
No vs. yes	POF	0.85	0.131	5.505	0.8643
No vs. yes	FOF	1	0.117	8.561	1.000

a NOF was the reference group throughout all the comparisons;

* p < 0.05.

*VFSS, videofluoroscopic swallowing study; NOF, non-oral feeding; OF, oral feeding; POF, partial oral feeding; FOF, full oral feeding*.

### Changes in VFSS Findings

**Figure 2:**
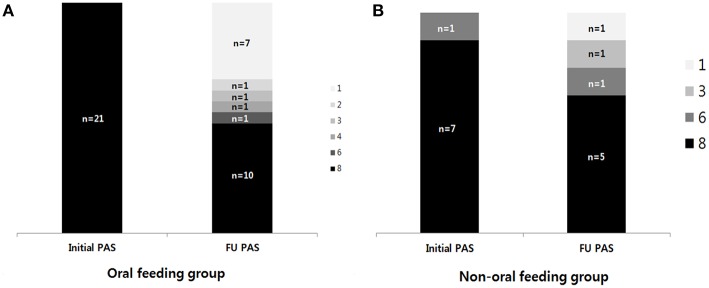
The change in the penetration-aspiration scale (PAS) between the first VFSS and follow-up VFSS. **(A)** Oral feeding group; **(B)** non-oral feeding group. (1) Material does not enter the airway. (2) Material enters the airway, remains above the vocal folds, and is ejected from the airway. (3) Material enters the airway, remains above the vocal folds, and is not ejected from the airway. (4) Material enters the airway, contacts the vocal folds, and is ejected from the airway. (5) Material enters the airway, contacts the vocal folds, and is not ejected from the airway. (6) Material enters the airway, passes below the vocal folds, and is ejected into the larynx or out of the airway. (7) Material enters the airway, passes below the vocal folds, and is not ejected from the trachea despite effort. (8) Material enters the airway, passes below the vocal folds, and no effort is made to eject.

For 29 children with follow-up VFSS (*n* = 21, OF group; *n* = 8, NOF group), changes in PAS and PTT were analyzed. The change in PAS between the first and follow-up VFSS according to each group is shown in Figure 2. The OF group showed a significant difference between the initial PAS (median 8, IQR: 8–8) and follow-up PAS (median 6, IQR: 1–8) (*p* = 0.003 for Wilcoxon signed-rank test, Figure 2A). However, there was no difference between the initial PAS (median PAS, IQR: 8–8) and follow-up PAS (median 8, IQR: 3.75–8) in the NOF group (*p* = 0.180 for Wilcoxon signed-rank test, Figure 2B).

In the OF group, there was a tendency for follow-up PTT (median 0.701 s, IQR: 0.600–0.984) to be shorter than the initial PTT (median 0.901s, IQR: 0.784–1.517), although the difference was not statistically significant (*p* = 0.077 for paired *t*-test). In the NOF group, follow-up PTT (median 1.418 s, IQR: 0.985–3.162) was not different from the initial PTT (median 1.437 s, IQR: 0.851–2.386). There was no difference in initial PTT between the OF and NOF groups (*p* = 0.153 for Mann-Whitney U-test), but the follow-up PTT was shorter in the OF group than in the NOF group (*p* = 0.003 for Mann-Whitney U-test).

## Discussion

There have been few reports regarding the effects of oral feeding trial on the development of swallowing function in children with aspiration. The results of this study suggest that swallowing function might improve over time if infants or young children attempt oral feeding before the aero-digestive centers are fully established despite aspiration findings on VFSS. Moreover, improvement in follow-up PAS was observed only in the group that received oral feeding.

From birth to 3 years of age, children learn to adapt the swallowing mechanism to accommodate different foods, fluids, and textures (11, 12). Ruark et al. (13) investigated pharyngeal muscle activity during swallowing in children, and showed that children established control of swallowing with different consistencies of food until 5 years of age. Even in children with aspiration who had undergone gastrostomy, it was reported that within a year more than half of the children showed improved swallowing function on VFSS when they were allowed oral feeding (3). However, the study primarily investigated the trajectory of deglutition function in children with gastrostomy and did not evaluate the effects of oral feeding challenge.

### The Impacts of Tracheostomy on Swallowing Function

In adults with stroke, it was reported that patients with tracheostomies had inferior swallowing function and kinematics compared to those without tracheostomies (14). In children with tracheostomies, it was reported that the time required to close the laryngeal vestibule once the arytenoids initiate anterior movement was longer than in those with no tracheostomy (15). Pre-swallow spillage and deficit in the pharyngeal phase were also frequently observed in children with tracheostomy (15).

Although tracheostomy interferes with swallowing function, it has the potential to promote oral feeding challenge (16). Oral feeding may be less likely to induce aspiration pneumonia because of the high accessibility of suctioning through tracheostomy. In this study, the oral feeding group showed better feeding outcome than the non-oral feeding group, suggesting the possibility of losing this opportunity if the feeding trial was not implemented in the infants or young children with tracheostomy.

### The Course of Children With Neuromuscular Disease

Although in the present study there was a difference in the frequency of neuromuscular disease between those who received oral feeding and those who did not, this seemed not to be a factor affecting feeding outcome after 1 year according to the multinomial logistic regression analysis. Among the OF group, 5 children were diagnosed with neuromuscular disease, 3 with congenital myopathy, and 2 with muscular dystrophy. Among them, 3 achieved FOF and 2 achieved POF after 1 year. Two children developed pneumonia within 1 year. Among the NOF group, 8 children were diagnosed with neuromuscular disease, 5 with congenital myopathy, 2 with muscular dystrophy, and 1 with motor neuron disease. Among them, 4 achieved POF and 4 remained at NOF status. Four of them developed pneumonia within 1 year. These results suggest that the swallowing function could be improved at least in the short term by the growth of the pharyngeal muscles (11, 17, 18) and longer duration of pharyngeal muscle activity (13) despite the progressive nature of neuromuscular disease, and oral feeding challenge might help attain the improvement.

### Concerns for Oral Feeding Trial in Children With Tracheostomy

Firstly, frequent suctioning may cause laryngeal wall irritation and tracheal wall injury. Injurious prolapse of the tracheal mucosa may occur by suctioning (19). Although there have been no guideline regarding the technical aspects of suctioning for this specific purpose, judicious gentle suctioning could be helpful in preventing those injuries on trachea.

Secondly, many parents of children who underwent an oral feeding trial reported that food was expelled through the tracheostomy during feeding without suctioning (for example, when the grape juice was fed, the purple liquid came out). Suction should be considered to be an incomplete method of removing all aspirates. If the aspirate not removed by suction or drainage through the tracheostomy exceeds a certain threshold, respiratory complications, such as aspiration pneumonia might occur. However, aspirates below a certain amount may not cause problems. Weiss (20) suggested that infants and children may lack some of the compensatory mechanisms that enable adults to protect the airway. Tutor and Gosa reported that aspiration was observed in children with non-pathologic conditions (21).

### Study Limitations

There are several limitations to this study. Firstly, this was a single-center study with a small number of subjects, which may have led to a selection bias. Secondly, because this study retrospectively obtained information from the medical records, it is possible that patients were regarded as having no respiratory complications if they had been previously treated for pneumonia in another hospital. Moreover, we could not clearly distinguish whether pneumonia was related to aspiration or not; thus, we analyzed pulmonary admission days and occurrence of pneumonia, as performed in the previous studies (3, 20, 22). Thirdly, although multinomial logistic regression analysis revealed that neuromuscular disease did not affect the feeding outcome, the proportion of patients with such disease was higher in the NOF group, which may have affected the outcome. Lastly, information regarding weight loss or dehydration that could be induced from oral feeding challenges was not obtained owing to the retrospective nature of the study. When the children experienced these problems, they also received tube feeding in parallel; hence, they were categorized into the POF group.

## Conclusions

In infants and young children with tracheostomy, oral feeding challenge improved the feeding outcome without an increased risk of pneumonia, although aspiration was confirmed by VFSS. These results suggest that oral feeding challenges might be attempted if aspirates can be removed through the tracheostomy even with the findings of aspiration.

## Data Availability

The datasets generated for this study are available on request to the corresponding author.

## Ethics Statement

All procedures performed in studies involving human participants were in accordance with the ethical standards of the institutional and/or national research committee and with the 1964 Helsinki declaration and its later amendments or comparable ethical standards. Ethical approval was obtained from the Seoul National University Hospital Institutional Review Board (IRB) No. 1803-115-932, which waived the requirement for informed consent due to the retrospective nature of the study.

## Author Contributions

YY: acquisition of data, analysis and interpretation of data, writing, and critical revision of manuscript. B-MO: analysis and interpretation of data, and critical revision of manuscript. SY: critical revision of manuscript. H-IS: study concept and design, acquisition of data, analysis and interpretation of data, study supervision, and critical revision of manuscript for intellectual content.

### Conflict of Interest Statement

The authors declare that the research was conducted in the absence of any commercial or financial relationships that could be construed as a potential conflict of interest.
